# Kidney Outcomes in Transthyretin Amyloid Cardiomyopathy

**DOI:** 10.1001/jamacardio.2024.4578

**Published:** 2024-11-17

**Authors:** Adam Ioannou, Yousuf Razvi, Aldostefano Porcari, Muhammad U. Rauf, Ana Martinez-Naharro, Lucia Venneri, Salsabeel Kazi, Ali Pasyar, Carina M. Luxhøj, Aviva Petrie, William Moody, Richard P. Steeds, Brett W. Sperry, Ronald M. Witteles, Carol Whelan, Ashutosh Wechalekar, Helen Lachmann, Philip N. Hawkins, Scott D. Solomon, Julian D. Gillmore, Marianna Fontana

**Affiliations:** 1 National Amyloidosis Centre, Royal Free Hospital, University College London, London, United Kingdom; 2 Center for Diagnosis and Treatment of Cardiomyopathies, Cardiovascular Department, Azienda Sanitaria Universitaria Giuliano-Isontina, University of Trieste, Trieste, Italy; 3 University College London, London, United Kingdom; 4 Department of Cardiology, Queen Elizabeth Hospital Birmingham, Birmingham, United Kingdom; 5 Institute of Cardiovascular Sciences, University of Birmingham, Edgbaston, Birmingham, United Kingdom; 6 Saint Luke’s Mid America Heart Institute, Kansas City, Missouri; 7 Stanford University School of Medicine, Stanford, California; 8 Cardiovascular Division, Brigham and Women’s Hospital, Harvard Medical School, Boston, Massachusetts

## Abstract

**Importance:**

Transthyretin amyloid cardiomyopathy (ATTR-CM) is a progressive cardiomyopathy that commonly presents with concomitant chronic kidney disease. Chronic kidney dysfunction is associated with worse outcomes, but the prognostic value of changes in kidney function over time has yet to be defined.

**Objective:**

To assess the prognostic importance of a decline in estimated glomerular filtration rate (eGFR) in a large cohort of patients with ATTR-CM.

**Design, Setting, and Participants:**

This retrospective, observational, single-center cohort study evaluated patients diagnosed with ATTR-CM at the National Amyloidosis Centre (NAC) in the UK who underwent an eGFR baseline assessment and a follow-up assessment at 1 year between January 2000 and April 2024. Data analysis was performed in June 2024.

**Main Outcomes and Measures:**

The primary outcome was the risk of all-cause mortality associated with decline in kidney function (defined as a decrease in eGFR >20%).

**Results:**

Among 2001 patients, mean (SD) age was 75.5 (8.4) years, and 263 patients (13.1%) were female. The median (IQR) change in eGFR was −5 mlL/min/1.73 m^2^ (−12 to 1), and 481 patients (24.0%) experienced decline in kidney function. Patients who experienced decline in kidney function more often had the p.(V142I) genotype than patients with stable kidney function (99 [20.6%] vs 202 [13.3%]; *P* < .001) and had a more severe cardiac phenotype at baseline, as evidenced by higher median (IQR) concentrations of serum cardiac biomarkers (N-terminal pro-B-type natriuretic peptide [NT-proBNP]: 2949 pg/mL [1759-5182] vs 2309 pg/mL [1146-4290]; *P* < .001; troponin T: 0.060 ng/mL [0.042-0.086] vs 0.052 ng/mL [0.033-0.074]; *P* < .001), while baseline median (IQR) kidney function was similar between the 2 groups (eGFR: 63 mL/min/1.73 m^2^ [51-77] vs 61 mL/min/1.73 m^2^ [49-77]; *P* = .41). Decline in kidney function was associated with a 1.7-fold higher risk of mortality (hazard ratio [HR], 1.71; 95% CI, 1.43-2.04; *P* < .001), with a similar risk across the 3 genotypes (wild type: HR, 1.64; 95% CI, 1.31-2.04; p.(V142I): HR, 1.70; 95% CI, 1.21-2.39; non-p.(V142I): HR, 1.51; 95% CI, 0.87-2.61) (*P* for interaction = .93) and the 3 NAC disease stages (stage 1: HR, 1.69; 95% CI, 1.22-2.32; stage 2: HR, 1.69; 95% CI, 1.30-2.18; stage 3: HR, 1.61; 95% CI, 1.11-2.35) (*P* for interaction = .97). Decline in kidney function remained independently associated with mortality after adjusting for increases in NT-proBNP and outpatient diuretic intensification (HR, 1.48; 95% CI, 1.23-2.76; *P* < .001).

**Conclusions and Relevance:**

In this retrospective cohort study, decline in kidney function was frequent in patients with ATTR-CM and was consistently associated with an increased risk of mortality, even after adjusting for established markers of worsening ATTR-CM. eGFR decline represents an independent marker of ATTR-CM disease progression that could guide treatment optimization in clinical practice.

## Key Points

**Question:**

What is the prognostic importance of a decline in kidney function in patients with transthyretin amyloid cardiomyopathy (ATTR-CM)?

**Findings:**

In this cohort study of 2001 patients, 481 patients (24.0%) experienced a decline in kidney function (defined as a decrease in estimated glomerular filtration rate by more than 20% at 1 year). Progression of kidney dysfunction was associated with a 1.7-fold higher risk of mortality, which was consistent across the 3 genotypes and disease stages and remained independently associated with mortality after adjusting for established markers of ATTR-CM disease progression.

**Meaning:**

Decline in kidney function in patients with ATTR-CM is frequent and represents an independent marker of disease progression.

## Teaser

This single-center cohort study assesses the prognostic importance of a decline in kidney function as assessed by estimated glomerular filtration rate in a large cohort of patients with transthyretin amyloid cardiomyopathy.

## Introduction

Transthyretin amyloid cardiomyopathy (ATTR-CM) is a progressive and ultimately fatal cardiomyopathy characterized by the deposition of misfolded transthyretin in the form of amyloid fibrils within the myocardial extracellular space, which disrupt cardiac structure and function.^1^ The sporadic, noninherited, wild-type ATTR-CM (wtATTR-CM) is a condition of older, predominantly male individuals, while the hereditary form of ATTR-CM (hATTR-CM) can present earlier in life with a varying clinical phenotype, often composed of both restrictive cardiomyopathy and polyneuropathy.^3^

In the setting of heart failure, chronic kidney disease (CKD) is frequent and associated with a higher risk of adverse outcomes, including heart failure hospitalizations and cardiovascular mortality.^5^ This premise extends to ATTR-CM and is most notably demonstrated in a well-established staging system that combines estimated glomerular filtration rate (eGFR) with N-terminal pro-B-type natriuretic peptide (NT-proBNP) to accurately stratify patients into prognostic categories.^7^ Worsening heart failure can influence kidney function through a wide array of interlinked mechanisms, including a decrease in kidney blood flow, venous congestion, impaired kidney hemodynamics, and neurohormonal overactivation. It is therefore conceivable that changes in kidney function could reflect changes in heart failure severity.^8^ However, despite the demonstrated link between heart failure and kidney function, the significance of changes in eGFR in patients with ATTR-CM has yet to be characterized. The aim of this study was to assess the prognostic importance of a decrease in eGFR as a marker of declining kidney function in a large cohort of patients with ATTR-CM.

## Methods

This is a retrospective, observational cohort study of patients diagnosed with ATTR-CM at the National Amyloidosis Centre (NAC) in the UK who underwent a baseline assessment and a follow-up assessment 1 year later between January 2000 and April 2024, with data analysis performed in June 2024. The diagnosis of ATTR-CM was established on the basis of validated diagnostic criteria,^9^ and patients who underwent an eGFR measurement at baseline and a follow-up measurement at 1 year were eligible for inclusion. GFR was estimated according to the standard Modification of Diet in Renal Disease formula (including the correction for race).^7^ All patients underwent genetic sequencing of the *TTR* gene and provided written consent for their data to be retrospectively analyzed and published, in line with the Declaration of Helsinki and approval from the Royal Free Hospital ethics committee (REC 21/PR/0620).

### Statistical Analysis

Statistical analysis was performed using Stata version 17 (StataCorp). All continuous variables were tested for normality using the Shapiro-Wilk test and are presented as means with standard deviations if the distribution was normal or as medians with interquartile ranges if otherwise, except for NT-proBNP, which was log-transformed for hypothesis testing. The independent-sample *t *test was used to compare means if the data were normally distributed in each group, and the nonparametric Mann-Whitney *U* test was used to compare the distributions of the 2 groups otherwise. Categorical data are presented as frequencies and percentages and compared using the χ^2^ test.

The associations between the change in eGFR, baseline characteristics, and other markers of ATTR-CM disease progression were assessed using linear regression, with the outcome variable being eGFR at 1 year and explanatory variables being baseline eGFR and either baseline characteristics, NT-proBNP progression (defined as an increase of >700 pg/mL and >30%), or outpatient diuretic intensification (defined as any initiation or increment in the dose of loop diuretic [furosemide equivalent]).^10^ (To convert NT-proBNP from pg/mL to ng/L, multiply by 1.) The regression coefficient for a given explanatory variable represented the estimated difference in mean eGFR at 1 year for a 1-unit change in the explanatory variable after adjusting for the baseline eGFR. All variables with a *P* value less than .10 were included in the multivariable analysis.

The optimal cut point for the percentage reduction in eGFR at 1 year was established using time-dependent receiver operating characteristic curves, followed by the Youden method. The optimal cut point was a reduction of 19.3% (sensitivity, 33.1%; specificity, 78.6%). A cut point of a reduction of 20% (sensitivity, 31.8%; specificity, 79.8%) was a good discriminator of survival by the log-rank test, and therefore decline in kidney function was defined as a decrease in eGFR of more than 20%.

Landmark survival analysis was carried out by assessing the association between decline in kidney function at the 1-year follow-up time point with all-cause mortality from that point onwards. Survival was evaluated using Cox proportional hazards regression analysis with estimated hazard ratios (HRs) with 95% confidence intervals. The proportional hazards assumption was checked and confirmed using weighted Schoenfeld residuals. Significant results were followed by internal validation of the model, which was achieved by performing a bootstrapping procedure with 500 repeats, allowing comparison of the percentile and bias-corrected methods to ensure the results were unbiased. Multivariable Cox regression models were adjusted for known predictors of mortality, and possible collinearity among candidate predictors was assessed using variance inflation factors with threshold equal to 5.

To assess the potential modification of effects across different baseline characteristics, additional models were created, each with an interaction term, and the significance from 0 of the coefficient associated with the interaction term was explicitly evaluated. Significant results were followed by sensitivity analyses to assess whether these results could be replicated using the CKD Epidemiology Collaboration (CKD-EPI) formula to estimate GFR.^11^

A second landmark analysis was composed of patients prescribed disease-modifying therapy for ATTR-CM or enrolled into clinical trials who underwent assessment at the start date and follow-up assessment at 1 year. This assessed the association between decline in kidney function at the 1-year follow-up time point with all-cause mortality from 1 year onwards.

The likelihood ratio test was used to evaluate the contribution of adding decline in kidney function to the model with established markers of ATTR-CM disease progression. The Akaike information criterion (AIC) and Harrell C statistic were calculated to measure the discriminatory ability of each model. The C statistics were compared by randomly dividing the dataset into 2 cohorts 1:1. The models were fitted to the first cohort, and the C statistics were compared in the second cohort using a *t* test after creating jackknife standard errors. Kaplan-Meier curves were constructed to view survival in different groups. Statistical significance was defined as *P* < .05 with 2-tailed tests.

## Results

### Baseline Characteristics

The study population was composed of 2001 patients, of whom 1385 (69.2%) had wtATTR-CM, 301 (15.0%) had p.(V142I) hATTR-CM, and 315 (15.7%) had non-p.(V142I) hATTR-CM. The mean (SD) age of the population was 75.5 (8.4) years, and 263 patients (13.1%) were female. The median (IQR) NT-proBNP concentration was 2466 pg/mL (1261-4533), the median (IQR) eGFR was 61 mL/min/1.73 m^2^ (50-77), and most of the population had NAC stage 1 (1078 patients [53.9%]) or NAC stage 2 (687 patients [34.3%]) disease.

At 1 year, 481 patients (24.0%) experienced a decline in kidney function (defined as a decrease in eGFR of >20%), while baseline kidney function was similar between those whose kidney function did and did not progress (Table; eTable 1 in Supplement 1). Patients who experienced decline in kidney function were more likely to have the p.(V142I) genotype and to have a more severe cardiac phenotype, as evidenced by a greater proportion of patients having New York Heart Association (NYHA) functional class III or IV and NAC stage 3 (severe) disease, driven by a higher median NT-proBNP and higher biomarker concentrations suggestive of hepatic congestion. β-Blockers, angiotensin-converting enzyme inhibitors (ACEIs)/angiotensin II receptor blockers (ARBs)/angiotensin receptor-neprilysin inhibitors (ARNIs), and mineralocorticoid receptor antagonist (MRA) medications were more commonly prescribed in patients who experienced decline in kidney function, and loop diuretics were prescribed at higher dosages. Echocardiographic assessments demonstrated that those experiencing kidney disease progression had a greater wall thickness and worse systolic and diastolic LV function (Table).

**Table.** Baseline Characteristics for Patients Diagnosed With Transthyretin Amyloid Cardiomyopathy (ATTR-CM) Who Subsequently Experienced Decline in Kidney Function (Defined as a Decrease in Estimated Glomerular Filtration Rate [eGFR] of >20%) Compared to Those Who Had Stable Kidney Function at 1 Year

Characteristic	No. (%)			*P* value
	Total population (N = 2001)	Stable kidney function (n = 1520 [76.0])	Decline in kidney function (n = 481 [24.0])	
Age, mean (SD), y	75.5 (8.4)	75.4 (8.8)	76.0 (7.5)	.55
Sex				
Female	263 (13.1)	201 (13.2)	62 (12.9)	.85
Male	1738 (86.9)	1319 (86.8)	419 (87.1)	
Race^a^				
Asian	30 (1.5)	19 (1.3)	365 (75.9)	.03
Black	377 (18.8)	272 (17.9)	105 (21.8)	
White	1594 (79.7)	1229 (80.9)	11 (2.3)	
Genotypes				
wtATTR	1385 (69.2)	1061 (69.8)	324 (67.4)	<.001
p.(V142I) hATTR	301 (15.0)	202 (13.3)	99 (20.6)	
Non-p.(V142I) hATTR^b^	315 (15.7)	257 (16.9)	58 (12.1)	
Baseline comorbidities				
Ischemic heart disease	365 (18.2)	265 (17.4)	100 (20.8)	.10
Diabetes	289 (14.4)	208 (13.7)	81 (16.8)	.09
Hypertension	629 (31.4)	468 (30.8)	161 (33.5)	.27
Stroke or TIA	192 (9.6)	153 (10.1)	39 (8.1)	.20
Atrial fibrillation or flutter	945 (47.2)	711 (46.8)	234 (48.7)	.47
CKD stage 3-5	925 (46.2)	712 (46.8)	213 (44.3)	.33
Heart failure severity				
NYHA class				
1	272 (13.6)	225 (14.8)	47 (9.8)	.003
2	1248 (62.4)	950 (62.5)	298 (62.0)	
3	387 (19.3)	275 (18.1)	112 (23.3)	
4	22 (1.1)	14 (0.9)	8 (1.7)	
Missing, No.	72	56	16	
Systolic blood pressure, mean (SD), mm Hg	123.1 (21.5)	123.4 (21.8)	122.4 (20.3)	.20
Diastolic blood pressure, mean (SD), mm Hg	74.9 (10.9)	75.0 (11.0)	74.4 (10.8)	.56
Heart rate, mean (SD), bpm	76.0 (19.3)	75.9 (20.0)	76.5 (18.5)	.15
Blood biomarkers				
NAC stage				
1	1078 (53.9)	851 (56.0)	227 (47.2)	.001
2	687 (34.3)	489 (32.2)	198 (41.2)	
3	236 (11.8)	180 (11.8)	56 (11.6)	
NT-proBNP, median (IQR), pg/mL	2466 (1261 to 4533)	2309 (1146 to 4290)	2949 (1759 to 5182)	<.001
eGFR, median (IQR), mL/min/1.73 m^2^	61 (50 to 77)	61 (49 to 77)	63 (51 to 77)	.41
Troponin T, median (IQR), ng/mL	0.053 (0.035 to 0.078)	0.052 (0.033 to 0.074)	0.060 (0.042 to 0.086)	<.001
Hemoglobin, median (IQR), g/dL	13.7 (12.6 to 14.8)	13.7 (12.7 to 14.8)	13.6 (12.2 to 14.6)	.002
Serum total bilirubin, median (IQR), mg/dL	0.76 (0.53 to 1.05)	0.70 (0.53 to 1.05)	0.82 (0.58 to 1.11)	.001
Alanine transaminase, median (IQR), U/L	25 (19 to 33)	25 (19 to 33)	26 (20 to 33)	.17
Alkaline phosphatase, median (IQR), U/L	90 (71 to 119)	88 (69 to 116)	98 (76 to 136)	<.001
GGT, median (IQR), U/L	66 (35 to 138)	61 (30 to 126)	84 (40 to 176)	<.001
Echocardiographic parameters, mean (SD)				
IVSd, mm	16.7 (2.6)	16.6 (2.6)	17.0 (2.5)	.006
PWTd, mm	16.3 (2.6)	16.2 (2.7)	16.5 (2.4)	.04
LVEF, %	48.7 (10.8)	49.1 (10.8)	47.6 (10.7)	.01
Longitudinal strain, %	−11.3 (3.8)	−11.4 (3.8)	−10.9 (3.6)	.04
E/e’	16.5 (7.1)	16.3 (7.2)	17.1 (6.7)	.001
Medications				
β-Blockers	1037 (51.8)	763 (50.2)	274 (57.0)	.01
ACEI/ARB/ARNI	1121 (56.0)	823 (53.8)	298 (62.0)	.003
MRA	766 (38.3)	539 (35.5)	227 (47.2)	<.001
SGLT2i	80 (4.0)	63 (4.1)	17 (3.5)	.55
Loop diuretic	1357 (67.8)	992 (65.3)	365 (75.9)	<.001
Daily furosemide equivalent dose, median (IQR), mg/kg	0.4 (0.0 to 0.7)	0.4 (0 to 0.6)	0.5 (0 to 0.7)	<.001

Abbreviations: ACEI, angiotensin-converting enzyme inhibitor; ARB, angiotensin receptor blocker; ARNI, angiotensin receptor-neprilysin inhibitor; bpm, beats per minute; CKD, chronic kidney disease; GGT, γ-glutamyl transferase; hATTR-CM, hereditary ATTR-CM; IVSd, interventricular septum in diastole; LVEF, left ventricular ejection fraction; MRA, mineralocorticoid receptor antagonist; NAC, National Amyloidosis Centre; NT-proBNP, N-terminal pro-B-type natriuretic peptide; NYHA, New York Heart Association; PWTd, posterior wall thickness in diastole; SGLT2i, sodium-glucose cotransporter-2 inhibitor; TIA, transient ischemic attack; wtATTR-CM, wild-type ATTR-CM.

SI conversion factors: To convert hemoglobin from g/dL to g/L, multiply by 10; NT-proBNP, from pg/mL to ng/L, multiply by 1; serum total bilirubin, from mg/dL to µmol/L, multiply by 17.104; troponin T, from ng/mL to ng/L, multiply by 1000.

^a^ Race reported by patients.

^b^ Patients in the non-p.(V142I) hATTR-CM subgroup had the following variants: p.(Thr80Ala) (n = 174), p.(Val50Met) (n = 35), p.(Ser97Tyr) (n = 19), p.(Ile127Val) (n = 12), p.(Gly67Val) (n = 7), p.(Ile88Leu) (n = 7), p.(Ala117Ser) (n = 5), p.(Glu62Asp) (n = 5), p.(Glu109Lys) (n = 5), p.(Ile127Phe) (n = 4), p.(Gly26Ser) (n = 3), p.(Gly67Arg) (n = 3), p.(Arg54Gly) (n = 2), p.(Glu74Gly) (n = 2), p.(Gly67Glu) (n = 2), p.(Phe53Val) (n = 2), p.(Ser43Asn) (n = 2), p.(Tyr134Cys) (n = 2), p.(Tyr89Phe) (n = 2), p.(Ala120Ser) (n = 1), p.(Asp58Tyr) (n = 1), p.(Asp58Val) (n = 1), p.(Cys30Gly) (n = 1), p.(Glu62Lys) (n = 1), p.(Glu74Gln) (n = 1), p.(Glu74Leu) (n = 1), p.(Glu74Lys) (n = 1), p.(Glu102Lys) (n = 1), p.(Glu109Gln) (n = 1), p.(Glu112Lys) (n = 1), p.(Gly73Ala) (n = 1), p.(Gly73Glu) (n = 1), p.(Gly77Arg) (n = 1), p.(His110Asp) (n = 1), p.(Ile93Val) (n = 1), p.(Ile104Ser) (n = 1), p.(Ile104Thr) (n = 1), p.(Phe64Leu) (n = 1), p.(Ser70Arg) (n = 1), p.(Tyr116Ser) (n = 1), and p.(Val40Ile) (n = 1).

### Characteristics Associated With Change in eGFR

At 1 year, the median (IQR) absolute change in eGFR was −5 mL/min/1.73 m^2^ (−12 to 1) and the median (IQR) percentage change was −8.0% (−19.6% to 1.3%). Patients with p.(V142I) hATTR-CM experienced a greater median (IQR) decline in eGFR than those with wtATTR-CM (−7 mL/min/1.73 m^2^ [−14 to −1] vs −5 mL/min/1.73 m^2^ [−11 to −1]; *P* = .003) and a greater median (IQR) decline than those with non-p.(V142I) hATTR-CM (−3 mL/min/1.73 m^2^ [−12 to 0]; *P* < .001). This association was maintained following adjustment for the baseline eGFR, whereby the mean difference in eGFR decline between patients with the p.(V142I) genotype and the remaining patients was −3.4 mL/min/1.73 m^2^ (95% CI, −4.7 to −2.2; *P* < .001). At 1 year, 667 patients (33.3%) experienced NT-proBNP progression (defined as an increase of >700 pg/mL and >30%). Patients who experienced NT-proBNP progression had a greater decline in eGFR than those with a stable NT-proBNP, whereby the mean difference in eGFR (adjusted for baseline eGFR) between the 2 groups was −3.4 mL/min/1.73 m^2^ (95% CI, −4.3 to −2.4; *P* < .001). At 1 year, 575 patients (28.7%) experienced outpatient diuretic intensification (ODI). Patients who experienced ODI had a greater decline in eGFR than those with a stable diuretic dosage, with an adjusted mean difference of −3.2 mL/min/1.73 m^2^ (95% CI, −4.2 to −2.2; *P* < .001). These variables, alongside log NT-proBNP at baseline and ACEI/ARB/ARNI use, remained independently associated with a decline in eGFR in a multivariable linear regression model (eTable 2 in Supplement 1), whereas changes in echocardiographic measures of LV wall thickness and function were not associated with change in eGFR (eAppendix and eTable 2 in Supplement 1).

### Survival

In the overall population, the mortality rate was 15.4 deaths per 100 person-years (py) (95% CI, 14.2-16.7), and a decline in eGFR at 1 year was associated with an increased risk of mortality (for every 5-mL/min/1.73 m^2^ decrease in eGFR: HR, 1.04; 95% CI, 1.00-1.08; *P* = .03; for every 5% decrease in eGFR: HR, 1.05; 95% CI, 1.02-1.07; *P* < .001). Patients who died during follow-up had a greater decline in eGFR at 1 year than those who survived, whereby the difference in mean eGFR (adjusted for baseline eGFR and follow-up time) between the 2 groups was −1.7 mL/min/1.73 m^2^ (95% CI, −2.8 to −0.5; *P* = .005).

#### Decline in Kidney Function

**Figure 1.**
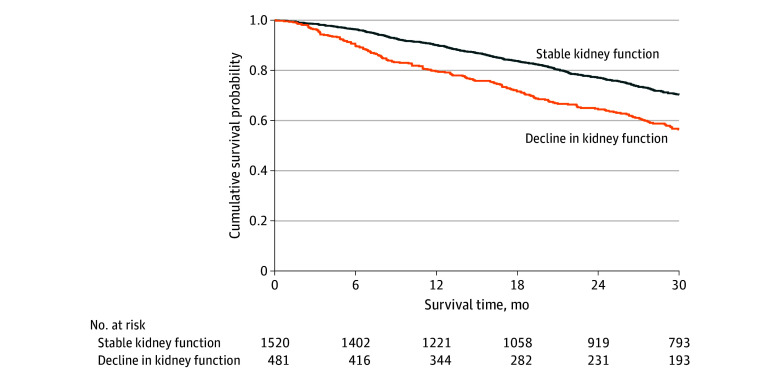
Kidney Progression and Survival Landmark Kaplan-Meier curves demonstrating the association between decline in kidney function at 1 year and subsequent survival.

Patients who experienced declining kidney function (defined as a decrease in eGFR of >20%) had a significantly higher death rate (22.6 deaths per 100 py; 95% CI, 19.5-26.1) than those who did not (13.4 deaths per 100 py; 95% CI, 12.1-14.8). Decline in kidney function was associated with a 1.7-fold higher risk of mortality (HR, 1.71; 95% CI, 1.43-2.04; *P* < .001) and the bootstrapped results indicated that the coefficients remained constant across the resamples, suggesting robustness in the association between declining kidney function and mortality (Figure 1). In a multivariable analysis adjusted for covariates including age, NAC disease stage, genotype, and NYHA class, decline in kidney function remained independently associated with mortality (HR, 1.61; 95% CI, 1.35-1.94; *P* < .001) (eTable 3 in Supplement 1).

**Figure 2.**
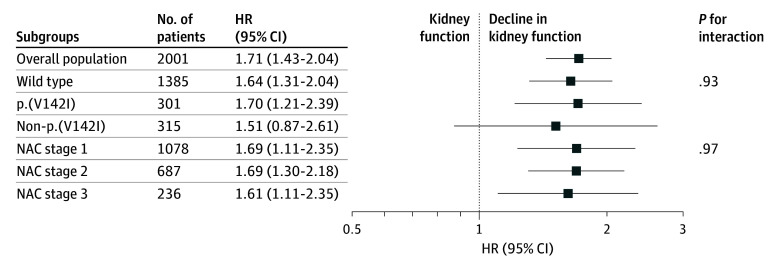
Risk of Mortality Associated With Decline in Kidney Function in Transthyretin Amyloid Cardiomyopathy The first *P* value for interaction is the interaction between decline in kidney function and genotypes and the second is the interaction between decline in kidney function and disease stages. HR indicates hazard ratio; NAC, National Amyloidosis Centre.

The risk of mortality associated with declining kidney function was consistent across the 3 genotypes (wtATTR-CM: HR, 1.64; 95% CI, 1.31-2.04; p.(V142I): HR, 1.70; 95% CI, 1.21-2.39; non-p.(V142I): HR, 1.51; 95% CI, 0.87-2.61) (*P* for interaction = .93) and the 3 NAC disease stages (stage 1: HR, 1.69; 95% CI, 1.22-2.32; stage 2: HR, 1.69; 95% CI, 1.30-2.18; stage 3: HR, 1.61; 95% CI, 1.11-2.35) (*P* for interaction = .97) (Figure 2). The risk of mortality associated with declining kidney function was also consistent regardless of sex (male: HR, 1.73; 95% CI, 1.44-2.09; female: HR, 1.57; 95% CI, 0.96-2.59) (*P* for interaction = .69), body mass index (*P* for interaction = .78), and whether patients had concomitant CKD (eGFR <60 mL/min/1.73 m^2^: HR, 1.80; 95% CI, 1.43-2.26; eGFR >60 mL/min/1.73 m^2^: HR, 1.74; 95% CI, 1.31-2.29) (*P* for interaction = .82) or diabetes (diabetes: HR, 1.88; 95% CI, 1.25-2.81; no diabetes: HR, 1.65; 95% CI, 1.36-2.01) (*P* for interaction = .54) and regardless of the 5-year period in which the baseline assessment occurred (*P* for interaction = .29). The risk associated with decline in kidney function in the overall population and subgroups remained consistent when the GFR was estimated according to the CKD-EPI formula (eTable 4 in Supplement 1).

In the study population, 461 patients (23.0%) were prescribed ATTR-CM disease-modifying therapy (10 patients were prescribed inotersen; 116, patisiran; 78, tafamidis; and 7, vutrisiran) or enrolled in clinical trials (n = 250), 347 of whom had assessments at the start date and 1 year after the start date (eFigure in Supplement 1). In this subgroup, 56 patients (16.1%) experienced declining kidney function 1 year after the initiation of disease-modifying therapy or enrollment in clinical trials. Patients who experienced declining kidney function had a higher death rate (14.2 deaths per 100 py; 95% CI, 8.3-23.9) than those who did not (6.1 deaths per 100 py; 95% CI, 4.3-8.6). Decline in kidney function was associated with a 2.4-fold higher risk of mortality (HR, 2.38; 95% CI, 1.26-4.46; *P* = .007), and the bootstrapped results indicated that the coefficients remained constant across the resamples, suggesting robustness in the association between decline in kidney function and mortality.

#### Combined Markers of Disease Progression

**Figure 3.**
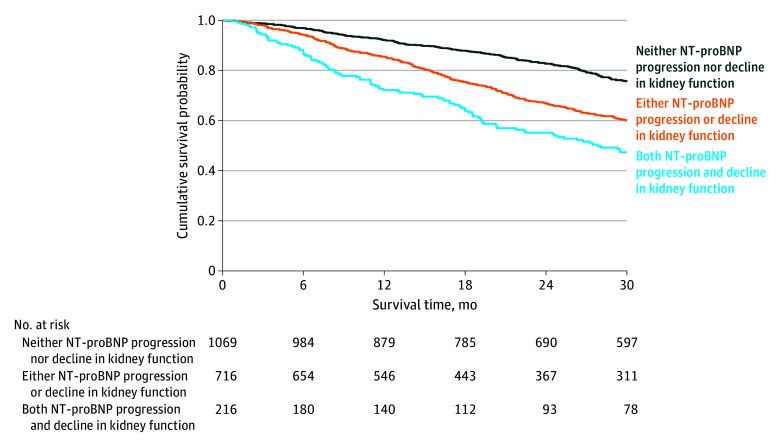
Combined Decline in Kidney Function and N-Terminal Pro-B-Type Natriuretic Peptide (NT-proBNP) Progression Landmark Kaplan-Meier curves demonstrating the association between decline in kidney function and NT-proBNP progression at 1 year and subsequent survival.

In a multivariable survival analysis, NT-proBNP progression (HR, 1.91; 95% CI, 1.61-2.26; *P* < .001) and decline in kidney function (HR, 1.55; 95% CI, 1.29-1.85; *P* < .001) were independently associated with mortality. The likelihood ratio test demonstrated that the addition of declining kidney function significantly improved the goodness of fit of the model (χ^2^, 22.08; *P* < .001) and significantly improved the discriminatory ability compared with a model composed of NT-proBNP progression alone (AIC: 8185 vs 8205; Harrell C statistic: 0.61; 95% CI, 0.57-0.64 vs 0.58; 95% CI, 0.55-0.61; *P* < .001). The increased risk associated with an incremental increase in markers of ATTR-CM disease progression at 1 year was similar across the 3 genotypes (*P* for interaction = .98) and the spectrum of NAC disease stages (*P* for interaction = .54) (Figure 3).

**Figure 4.**
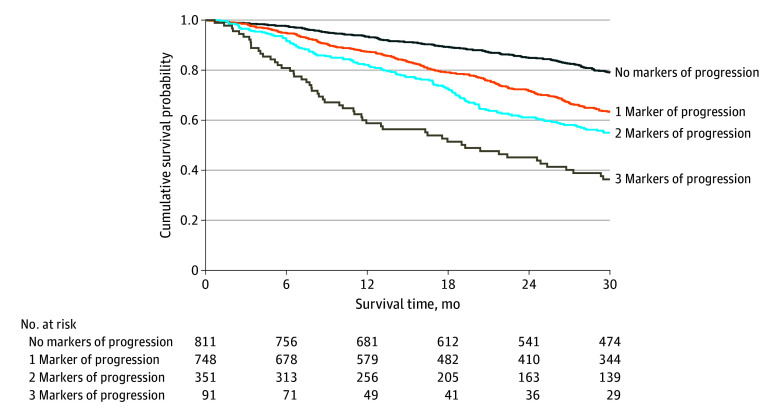
Combined Decline in Kidney Function, N-Terminal Pro-B-Type Natriuretic Peptide (NT-proBNP) Progression, and Outpatient Diuretic Intensification Landmark Kaplan-Meier curves demonstrating the association between markers of disease progression at 1 year and subsequent survival.

In a second multivariable survival analysis, NT-proBNP progression (HR, 1.85; 95% CI, 1.57-2.19; *P* < .001), ODI (HR, 1.61; 95% CI, 1.36-1.90; *P* < .001), and decline in kidney function (HR, 1.48; 95% CI, 1.23-2.76; *P* < .001) were independently associated with mortality. The likelihood ratio test demonstrated that the addition of declining kidney function significantly improved the goodness of fit of the model (χ^2^, 17.29; *P* < .001) and significantly improved the discriminatory ability compared with a model comprised of NT-proBNP progression and ODI (AIC: 8158 vs 8174; Harrell C statistic: 0.63; 95% CI, 0.59-0.66 vs 0.61; 95% CI, 0.58-0.64; *P* = .003). Each incremental increase in ATTR-CM progression markers was associated with an increased risk of mortality that was consistent across the 3 genotypes (*P* for interaction = .79) and the spectrum of NAC disease stages (*P* for interaction = .51) (Figure 4; eTable 5 in Supplement 1).

## Discussion

To our knowledge, this is the first study to comprehensively evaluate the importance of a change in kidney function in a large population of patients with ATTR-CM. The current data indicate the following: (1) decline in kidney function (defined as a decrease in eGFR of >20%) was frequent (approximately 1 in 4 patients) and was more common in patients with the p.(V142I) genotype and more advanced cardiac disease; (2) decline in kidney function was independently associated with an increased risk of mortality; and (3) combining declining kidney function with established markers of ATTR-CM disease progression enables further refinement of risk and could be highly relevant both in clinical practice to stratify disease progression and as an end point in clinical trials.

ATTR-CM is a progressive cardiomyopathy and is increasingly recognized as a multifaceted disease process.^12^ The underlying pathophysiology encompasses a complex interplay between the heart and kidneys, resulting in type 2 cardiorenal syndrome. However, the rate of decline in kidney function is extremely variable, likely reflecting the complex nature of ATTR-CM. Determinants of declining kidney function are multifactorial and depend on the underlying genotype, comorbidities, and cardiac disease severity—hence, there is a need to define the disease course and stratify progression at an individual patient level.^7^ Nevertheless, the rate of eGFR decline observed in this cohort appears greater than that observed in the general heart failure population, possibly reflecting the more severe and rapidly advancing nature of ATTR-CM, which is potentially compounded by the direct impact of kidney ATTR fibril deposition.^8^

Creatinine-based eGFR is the most widely used focal metric for assessing kidney function and forms an integral component of the standard heart failure assessment. The current study demonstrates that decline in kidney function occurs in approximately one-quarter of patients with ATTR-CM at 1 year. Patients with p.(V142I) hATTR-CM experienced a more rapid decline in eGFR than those with wtATTR-CM and non-p.(V142I) hATTR-CM. The p.(V142I) genotype is associated with a more aggressive and rapidly progressive cardiomyopathy, and it is likely that accelerated progression of cardiac disease results in a greater decline in kidney function.^14^ Patients who experienced decline in kidney function also had more advanced cardiac disease at baseline and were more commonly prescribed heart failure medications. Although renin-angiotensin system blockers and loop diuretics influence glomerular filtration, it is likely that prescription of these medications reflects a more advanced cardiac phenotype and that the rate of cardiac disease progression is the main driver of eGFR decline.^16^ This notion is supported by the observation that eGFR decline remained independently associated with established markers of cardiac disease progression (NT-proBNP progression and ODI) even after adjusting for comorbidities, such as diabetes, and heart failure medications.^10^ However, it is noteworthy that changes in echocardiographic measures of LV wall thickness and LV function were not associated with decline in kidney function.

Renin-angiotensin-aldosterone system inhibition through use of ACEI/ARB/ARNI medications and MRAs, along with sodium-glucose cotransporter 2 inhibitors (SGLT2i), have all demonstrated reno-protective properties.^17^ Considering the independent association between decline in kidney function and mortality, it is plausible that treatment with these medications could have a similar impact in ATTR-CM and may in part explain the reduced risk of mortality observed in patients treated with MRAs and SGLT2i.^16^

The current study, which represents the largest analysis of kidney outcomes in ATTR-CM, demonstrated that declining kidney function was associated with a 1.7-fold higher risk of mortality, with similar increased risk observed across the 3 genotypes and the spectrum of NAC disease stages. The risk associated with decline in kidney function was consistent regardless of whether patients had concomitant CKD or diabetes, suggesting that deterioration in eGFR reflects amyloid disease progression rather than the presence or severity of other comorbidities. Decline in kidney function was also associated with increased risk of mortality in patients initiated on ATTR-CM disease-modifying therapy or enrolled in clinical trials, although there is hope that disease-modifying therapy may attenuate eGFR decline.^21^

A novel definition of decline in kidney function in patients with ATTR-CM is of great importance. In the context of heart failure, declining kidney function is influenced by a multitude of factors and can fluctuate depending on fluid status, coprescribed heart failure medications, and neurohormonal changes that influence the cardiorenal axis.^5^ Therefore, a minimal clinically important difference in eGFR at an individual level is needed to define whether patients are experiencing a significant deterioration in kidney function.

Considering the rapidly evolving therapeutic landscape, there is an increasing clinical need to define markers of ATTR-CM disease progression that might identify the need to switch to alternative agents with different mechanisms of action or consider combination therapy.^10^ Serial echocardiographic assessments lack the precision to track disease progression. Although worsening stroke volume and valvular regurgitation are associated with mortality, these parameters are challenging to quantify and are subject to significant intraobserver and interobserver variability.^25^ Cardiac magnetic resonance with multiparametric mapping has demonstrated utility in tracking treatment response in cardiac light-chain amyloidosis, but this imaging modality is costly and only available in highly specialized centers.^26^ In contrast, the definition of decline in kidney function used in this study represents a simple, widely available, universally applicable marker of disease progression that could easily be applied in clinical practice to guide optimization of treatment strategies. Kidney outcomes could also be incorporated into contemporary clinical trials of ATTR-specific disease-modifying therapies. The inclusion of decline in kidney function as an end point may lead to an important increment in event rates, which could in turn influence contemporary trial design, with fewer patients and a reduced follow-up duration required to evaluate the efficacy of novel agents.^15^

Although eGFR decline was associated with established markers of ATTR-CM disease progression, all 3 markers of disease progression (decline in kidney function, NT-proBNP progression, and ODI) remained independently associated with mortality, indicating that each marker captures a slightly different aspect of the underlying disease progress. NT-proBNP elevation reflects a combination of pathophysiologic biochemical processes occurring in response to cardiac amyloid infiltration.^7^ ODI acts as a surrogate marker of worsening heart failure symptoms secondary to fluid accumulation, and declining kidney function reflects a deterioration of the cardiorenal axis whereby reduced cardiac output, venous congestion, and renin-angiotensin overactivation all contribute to a reduction in glomerular filtration.^28^ These routinely measured biomarkers could easily be combined to form a simple, universally applicable progression score that would refine the risk of mortality beyond each individual marker of progression.

### Limitations

This study’s results should be considered in the context of its limitations. There is a survival bias in that this study only included patients with follow-up 1 year after the baseline eGFR measurement. Therefore, the extent of differences between those with and without decline in kidney function may be underestimated, as rapid disease progression may have resulted in death before the follow-up assessment. This is a single-center study and therefore requires external validation. Finally, a minority of patients in this study were receiving disease-modifying therapies, and the thresholds and associations with mortality risk of biomarker progression may not be the same in other populations.

## Conclusions

In this large cohort of patients with ATTR-CM, decline in kidney function was common and consistently associated with an increased risk of mortality, even after adjustment for established markers of cardiac disease progression. The inclusion of declining kidney function as an end point in contemporary trials could allow the capture of more clinically meaningful and potentially modifiable events at earlier stages of disease. Combining decline in kidney function with established markers of cardiac disease progression (NT-proBNP progression and ODI) produces a simple, universally applicable model that enables further refinement of risk by encompassing multiple aspects of the cardiorenal axis in a single scoring system and thus detects patients experiencing rapid ATTR-CM progression who are at the highest risk of mortality.

## Notes

Supplement 1. **eAppendix.** eResults **eFigure.** CONSORT Diagram **eTable 1.** Chronic Kidney Disease Stage for Patients Diagnosed With ATTR-CM Who Subsequently Experienced Decline in Kidney Function Compared to Those Who Had a Stable Kidney Function at 1-Year **eTable 2.** Linear Regression Analysis Assessing the Association Between Characteristics and Change in eGFR **eTable 3.** Multivariable Cox Regression Analysis **eTable 4.** Univariable Cox Regression Analysis Using the Chronic Kidney Disease Epidemiology Collaboration (CKD-EPI) Formula to Estimate GFR **eTable 5.** Univariable Cox Regression Analysis Demonstrating the Risk of Mortality Associated With Different Combinations of Markers of ATTR-CM Progression

Supplement 2. Data Sharing Statement
