# Supplementary material for: Kidney Outcomes in Transthyretin Amyloid Cardiomyopathy
Source: JAMA Cardiol. 2024 Nov 17:e244578. Online ahead of print. doi: 10.1001/jamacardio.2024.4578 (PMC11571068; doi:10.1001/jamacardio.2024.4578)
Supplement: Supplement 1. — eAppendix. eResults eFigure. CONSORT Diagram eTable 1. Chronic Kidney Disease Stage for Patients Diagnosed With ATTR-CM Who Subsequently Experienced Decline in Kidney Function Compared to Those Who Had a Stable Kidney Function at 1-Year eTable 2. Linear Regression Analysis Assessing the Association Between Characteristics and Change in eGFR eTable 3. Multivariable Cox Regression Analysis eTable 4. Univariable Cox Regression Analysis Using the Chronic Kidney Disease Epidemiology Collaboration (CKD-EPI) Formula to Estimate GFR eTable 5. Univariable Cox Regression Analysis Demonstrating the Risk of Mortality Associated With Different Combinations of Markers of ATTR-CM Progression [file jamacardiol-e244578-s001.pdf]

Supplemental Online Content

Ioannou A, Razvi Y, Porcari A, et al. Kidney outcomes in transthyretin amyloid cardiomyopathy. *JAMA Cardiol*. Published online November 17, 2024. doi:10.1001/jamacardio.2024.4578

eAppendix. eResults
eFigure. CONSORT Diagram
eTable 1. Chronic Kidney Disease Stage for Patients Diagnosed With ATTR-CM Who Subsequently Experienced Decline in Kidney Function Compared to Those Who Had a Stable Kidney Function at 1-Year
eTable 2. Linear Regression Analysis Assessing the Association Between Characteristics and Change in eGFR
eTable 3. Multivariable Cox Regression Analysis
eTable 4. Univariable Cox Regression Analysis Using the Chronic Kidney Disease Epidemiology Collaboration (CKD-EPI) Formula to Estimate GFR
eTable 5. Univariable Cox Regression Analysis Demonstrating the Risk of Mortality Associated With Different Combinations of Markers of ATTR-CM Progression

This supplemental material has been provided by the authors to give readers additional information about their work.

© 2024 Ioannou et al. *JAMA Cardiology.*

eAppendix. eResults

There was a reduction in median eGFR from baseline to 1-year in the overall study population (61ml/min/m^2 [50 ml/min/1.73m^2 -77 ml/min/1.73m^2] vs 55ml/min/m^2[43 ml/min/1.73m^2 -70 ml/min/1.73m^2], P<0.001). There was also a significant reduction in median eGFR in patients who did not achieve the threshold used to define decline in kidney function (from 61ml/min/m^2 [50-77] to 59ml/min/m^2 [48 -74], P<0.001), but this was more evident in patients who manifested decline in kidney function (from 63ml/min/m^2 [51 -77] to 43ml/min/m^2 [34-54], P<0.001).

To assess whether decline in kidney function remained independently associated with mortality after adjusting for changes in echocardiographic parameters observed at 1-year an additional multivariable Cox regression analysis was carried out. This adjusted for the following covariates: change in interventricular septal thickness in diastole, change in left ventricular ejection fraction, change in E/e' and change in longitudinal strain. This analysis demonstrated that renal progression remained independently associated with an increased risk of mortality (HR=1.43, 95%CI[1.05-1.94], P=0.023).

To assess whether the year of baseline assessment influenced the prognostic importance of decline in kidney function, the study population was divided into 5 separate cohorts based on the year of the baseline assessment (cohort 1: 2000-2004, cohort 2: 2005-2009, cohort 3: 2010-2014, cohort 4: 2015-2019, cohort 5: 2020-2023). The risk associated with decline in kidney function was consistent across each time period (P for interaction = 0.288). Decline in kidney function also was independently associated with increased risk of mortality after adjusting for the time period in which the baseline assessment was performed (HR=1.72, 95%CI[1.43-2.05], P<0.001).

To assess whether body mass index (BMI) at baseline influenced the prognostic importance of decline in kidney function, the study population was divided into patients who were underweight (BMI <18.5kg/m^2), normal weight (BMI: 18.5 – 24.9kg/m^2), overweight (BMI: 25 – 29.9kg/m^2) and obese (BMI >30kg/m^2). The risk associated with decline in kidney function was consistent across each BMI category (P for interaction = 0.784).

ATTR-CM disease progression has previously been stratified by assessing the increase in NAC disease stage at 1-year. In the study population, 479 patients demonstrated an increase in NAC disease stage; this was associated with a 1.7-fold increased risk of mortality (HR=1.73, 95%CI[1.46-2.07], P<0.001). In a multivariable analysis adjusting for the increase in NAC disease stage, NT-proBNP progression and decline in kidney function , both NT-proBNP progression and declining kidney function remained independently associated with increased risk of mortality (Increase in NAC disease stage: HR=1.16, 95%CI[0.95-1.42], P=0.154; NT-proBNP progression: HR=1.49, 95%CI[1.23-1.80], P<0.001; decline in kidney function: HR=1.49, 95%CI[1.24-1.80], P<0.001).

Within the study population, 459 patients had measurement of troponin-T both at baseline and at 1 year. Increases in troponin-T was previously defined as an absolute increase >10ng/L and a percentage increase >20%. In the study population, an increase in troponin-T was associated with a 1.7-fold higher risk of mortality (HR=1.66, 95%CI[1.18-2.34], P=0.004) and in a multivariable Cox regression analysis both troponin-T (HR=1.60, 95%CI[1.13-2.26] P=0.008) and decline in kidney function (HR=1.48, 95%CI[1.03-2.14], P=0.036) remained independently associated with mortality.

© 2024 Ioannou et al. *JAMA Cardiology.*

eFigure. CONSORT Diagram

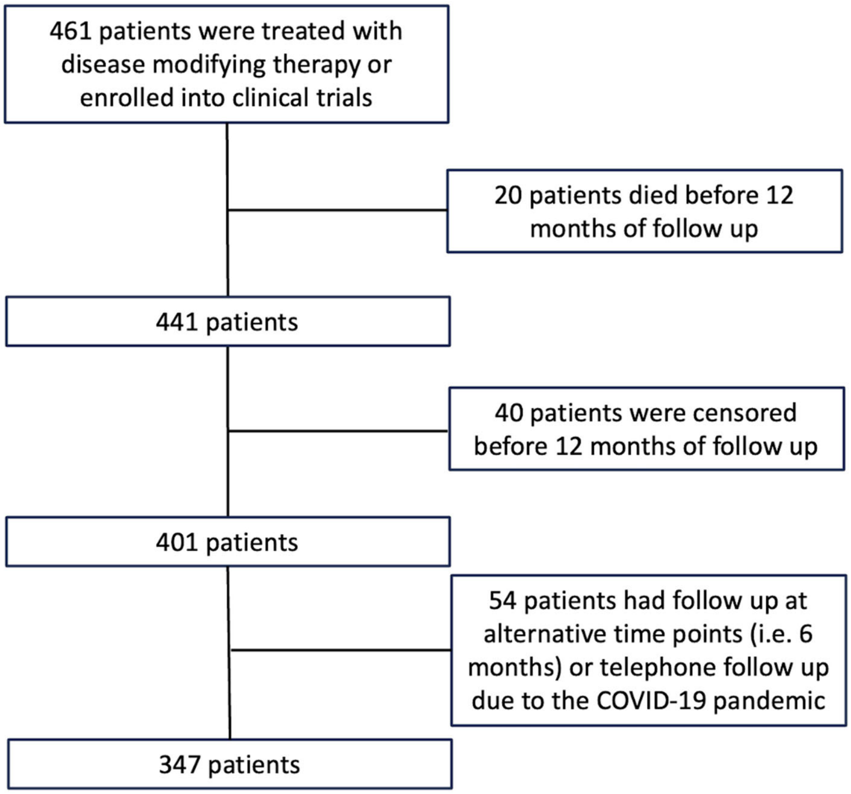

Consort diagram demonstrating which patients who were prescribed ATTR-CM disease modifying therapy or enrolled into clinical trials had a repeat assessment 1-year after their start date.

© 2024 Ioannou et al. *JAMA Cardiology.*

Chronic kidney disease stage	Total population (n = 2001)	Stable kidney function (n = 1520, 76.0%)	Decline in kidney function (n = 481, 24.0%)	P value
eGFR 60-90ml/min/m^2	1076 (53.8%)	808 (53.2%)	268 (55.7%)	0.542
eGFR 30-59ml/min/m^2	854 (42.7%)	656 (43.2%)	198 (41.2%)	
eGFR 15-29ml/min/m^2	67 (3.4%)	52 (3.4%)	15 (3.1%)	
eGFR <15ml/min/m^2	4 (0.2%)	4 (0.3%)	0 (0.0%)	

eTable 1. Chronic Kidney Disease Stage for Patients Diagnosed With ATTR-CM Who Subsequently Experienced Decline in Kidney Function Compared to Those Who Had a Stable Kidney Function at 1-Year

© 2024 Ioannou et al. *JAMA Cardiology.*

	Univariable linear regression adjusted for baseline eGFR		Multivariable linear regression adjusted for baseline eGFR	
**eGFR at 1 year**	**Coefficient (95% CI)**	**P-value**	**Coefficient (95% CI)**	**P-value**
Age (years)	-0.1 (-0.2 to -0.0)	0.001	-0.0 (-0.1 to 0.0)	0.142
Sex (male)	0.2 (-1.1 to 1.6)	0.719	-	-
Diabetes mellitus	-1.6 (-2.9 to -0.3)	0.017	-0.9 (-2.2 to 0.4)	0.157
p.(V142I) hATTR-CM	-3.4 (-4.7 to -2.2)	<0.001	-2.4 (-3.7 to -1.1)	<0.001
Log NT-pro-BNP (ng/L)	-2.4 (-2.9 to -1.9)	<0.001	-1.8 (-2.3 to -1.2)	<0.001
Haemoglobin (g/L)	0.0 (0.0 to 0.1)	0.014	0.0 (-0.0 to 0.0)	0.357
Beta-blocker	-1.2 (-2.1 to -0.2)	0.015	0.2 (-0.7 to 1.2)	0.614
ACEi/ARB/ARNI	-2.4 (-3.4 to -1.5)	<0.001	-1.5 (-2.5 to -0.6)	0.002
MRA	-1.9 (-2.8 to -0.9)	<0.001	-0.9 (-1.8 to 0.2)	0.098
Loop diuretics	-2.4 (-3.4 to -1.4)	<0.001	-0.6 (-1.6 to 0.5)	0.286
NT-proBNP progression	-3.4 (-4.3 to -2.4)	<0.001	-2.6 (-3.6 to -1.6)	<0.001
Outpatient diuretic intensification	-3.2 (-4.2 to -2.2)	<0.001	-2.3 (-3.3 to -1.3)	<0.001
Change in IVSd (mm)	0.0 (-0.3 to 0.4)	0.816	-	-
Change in LVEF (%)	0.0 (-0.0 to 0.1)	0.227	-	-
Change in E/e’	0.1 (-0.0 to 0.1)	0.131	-	-
Change in longitudinal strain (%)	-0.1 (-0.3 to 0.1)	0.213	-	-

eTable 2. Linear Regression Analysis Assessing the Association Between Characteristics and Change in eGFR

Linear regression analysis adjusted for the baseline eGFR, whereby each regression coefficient represents the adjusted mean difference in eGFR at 1 year for a unit increase in the variable of interest, followed by a multivariable linear regression analysis, which included the baseline eGFR and all variables with a P<0.10. Outpatient diuretic intensification was defined as any initiation or increment in the dose of loop diuretic.

© 2024 Ioannou et al. *JAMA Cardiology.*

eTable 3. Multivariable Cox Regression Analysis

Multivariable Cox regression analysis		
**Variables**	**HR (95%CI)**	**P value**
Age (years)	1.03 (1.02 – 1.04)	<0.001
Wild-type	Ref	
p.(V142I)	1.77 (1.44 – 2.18)	<0.001
Non-p.(V142I)	1.43 (1.07 – 1.93)	0.017
NAC stage 1	Ref	
NAC stage 2	2.07 (1.70 – 2.53)	<0.001
NAC stage 3	3.82 (3.02 – 4.84)	<0.001
NYHA class I	Ref	
NYHA class II	1.86 (1.28 – 2.70)	0.001
NYHA class III	2.26 (1.52 – 3.36)	<0.001
NYHA class IV	2.59 (1.31 – 5.10)	0.006
Decline in kidney function	1.61 (1.35 – 1.94)	<0.001

© 2024 Ioannou et al. *JAMA Cardiology.*

eTable 4. Univariable Cox Regression Analysis Using the Chronic Kidney Disease Epidemiology Collaboration (CKD-EPI) Formula to Estimate GFR

Subgroup	HR (95%CI)	P-value	P for interaction
Overall population	1.73 (1.46 – 2.04)	<0.001	-
Males	1.72 (1.44 – 2.06)	<0.001	0.884
Females	1.79 (1.12 – 2.86)	0.015	
Wild type	1.67 (1.35 – 2.06)	<0.001	0.966
p.(V142I)	1.63 (1.17 – 2.28)	0.004	
Non-p.(V142I)	1.56 (0.91 – 2.68)	0.103	
NAC stage 1 disease	1.55 (1.15 – 2.10)	0.004	0.862
NAC stage 2 disease	1.73 (1.35 – 2.22)	<0.001	
NAC stage 3 disease	1.62 (1.14 – 2.29)	0.006	
Diabetes	1.65 (1.10 – 2.42)	0.015	0.851
No diabetes	1.72 (1.43 – 2.08)	<0.001	
eGFR <60ml/min/m^2	1.78 (1.43 – 2.20)	<0.001	0.670
eGFR >60ml/min/m^2	1.65 (1.26 – 2.16)	<0.001	

© 2024 Ioannou et al. *JAMA Cardiology.*

eTable 5. Univariable Cox Regression Analysis Demonstrating the Risk of Mortality Associated With Different Combinations of Markers of ATTR-CM Progression

**Risk of mortality associated with different combinations of progression markers**		
	**HR (95%CI)**	**P value**
Decline in kidney function + NT-proBNP progression vs. Renal progression + ODI	0.90 (0.57 – 1.41)	0.635
Decline in kidney function + ODI vs. NT-proBNP + ODI	1.29 (0.83 – 1.99)	0.256
Decline in kidney function + NT-proBNP progression vs. NT-proBNP progression + ODI	1.15 (0.78 – 1.69)	0.471

© 2024 Ioannou et al. *JAMA Cardiology.*
