# Supplementary material for: Kidney Outcomes in Transthyretin Amyloid Cardiomyopathy
Source: JAMA Cardiol. 2024 Nov 17:e244578. Online ahead of print. doi: 10.1001/jamacardio.2024.4578 (PMC11571068; doi:10.1001/jamacardio.2024.4578)
Supplement: Supplement 2. — Data Sharing Statement [file jamacardiol-e244578-s002.pdf]

Data Sharing Statement

Ioannou. Kidney Outcomes in Transthyretin Amyloid Cardiomyopathy. *JAMA Cardiol*.
Published November 17, 2024. doi:10.1001/jamacardio.2024.4578

Data
Data available: No
